# Impact of sleep disturbance on patients in treatment for mental disorders

**DOI:** 10.1186/1471-244X-12-179

**Published:** 2012-10-29

**Authors:** Håvard Kallestad, Bjarne Hansen, Knut Langsrud, Torleif Ruud, Gunnar Morken, Tore C Stiles, Rolf W Gråwe

**Affiliations:** 1 Division of Psychiatry, Department of Research and Development, St. Olav’s University Hospital, Trondheim, Norway; 2 Faculty of Medicine, Department of Neuroscience, Norwegian University of Science and Technology, Trondheim, Norway; 3 Division of Psychiatry, Department of Østmarka, St. Olav’s University Hospital, Trondheim, Norway; 4 Division of Mental Health Services, Akershus University Hospital, Oslo, Norway; 5 Faculty of Medicine, Institute of Clinical Medicine, University of Oslo, Oslo, Norway; 6 Faculty of Social Sciences and Technology Management, Department of Psychology, Norwegian University of Science and Technology, Trondheim, Norway; 7 Institute of Clinical Medicine, Norwegian Centre for Addiction Research, University of Oslo, Oslo, Norway; 8 Drug and Alcohol Treatment Health Trust Central Norway, Department of Research and Development, Trondheim, Norway

## Abstract

**Background:**

In clinical practice, sleep disturbance is often regarded as an epiphenomenon of the primary mental disorder. The aim of this study was to test if sleep disturbance, independently of primary mental disorders, is associated with current clinical state and benefit from treatment in a sample representative of public mental health care clinics.

**Method:**

2246 patients receiving treatment for mental disorders in eight public mental health care centers in Norway were evaluated in a cross-sectional study using patient and clinician reported measures. Patients reported quality of life, symptom severity, and benefit from treatment. Clinicians reported disorder severity, level of functioning, symptom severity and benefit from treatment. The hypothesis was tested using multiple hierarchical regression analyses.

**Results:**

Sleep disturbance was, adjusted for age, gender, time in treatment, type of care, and the presence of any primary mental disorder, associated with lower quality of life, higher symptom severity, higher disorder severity, lower levels of functioning, and less benefit from treatment.

**Conclusion:**

Sleep disturbance ought to be considered a stand-alone therapeutic entity rather than an epiphenomenon of existing diagnoses for patients receiving treatment in mental health care.

## Background

Sleep disturbance affects 50% to 80% of all patients with mental disorders and it is currently a symptom of 19 axis I disorders [1-3]. At the same time, it is considered to be a disorder in itself if the sleep disturbance impairs daily functioning [4,5]. With this diagnostic multitude, there is a possibility that clinicians regard the sleep disturbance as an epiphenomenon that will be dissolved once the primary mental disorder is treated and not as a valid stand-alone clinical entity [1]. This distinction can have consequences for choice of treatment for these patients [1] and sleep disturbance is poorly recognized when patients have a mental disorder [6,7].

The relationship between sleep and mental disorders is complex and not fully understood. Sleep disturbance may precede depression [8-10], and 40% to 70% of patients who are successfully treated for depression experience sleep disturbance as a residual symptom [11-13]. On the other hand, the remission rate following anti-depressive treatment can be doubled if adjunct treatment for sleep disturbance is provided [14], and depression can be treated using cognitive behavior therapy for insomnia alone [15]. These findings challenge the assumption that the sleep disturbance is secondary to a primary disorder. It may be better conceptualized as a comorbid condition, at least in depression. In a state-of-the-science statement the National Institutes of Health (NIH) recommended that when insomnia occurs concurrent with other disorders it should be considered comorbid rather than secondary [16]. In the online draft for the DSM-5, this recommendation is taken into consideration and a paradigm shift is proposed as to how sleep disturbance should be conceptualized in patients with mental disorders [17]. It is suggested that insomnia should always be coded if the criteria are fulfilled, regardless of meeting criteria for other disorders.

Most research so far on the association between sleep disturbance and mental disorders have been conducted in selected patient groups, mostly depressed patients, that may not fully resemble the heterogeneous group of patients found in public health care systems. The clinical usefulness of the NIH recommendation to regard insomnia as a comorbid disorder, rather than an epiphenomenon to a primary mental disorder, would be further supported if it was demonstrated that sleep disturbance is associated with distress and disability independently of the patients’ primary diagnosis also in settings representative of mental health care. Studies investigating such associations may be particularly relevant now with the advent of the DSM-5 proposal. However, no studies have been conducted to test if sleep disturbance is associated with current clinical state and benefit from treatment for patients representative of clinical settings. The aim of the current study was thus to test the hypothesis that sleep disturbance, independently of the patients’ primary mental disorder, is associated with variations in quality of life, disorder and symptom severity, level of functioning, and benefit from treatment in a large, heterogenous, clinical sample.

## Method

### Procedure

The data in this cross-sectional study were collected from eight general mental health care centers during eight weeks in 2002 and four weeks in 2005. The data collections were commissioned by the Norwegian Department of Health and conducted by an independent research institution, SINTEF Technology and Society. One person was in charge of organizing the data collection from both patients and clinicians at each center. The centers were selected to be demographically representative for the country and the catchment areas of the included clinics covered about 10% of the Norwegian population in both urban and rural areas in different regions of the country. The assessments were the same across all sites and in both years. The two data-sets were pooled into one for the current study.

### Participants

All 6538 patients receiving treatment in the mental health care centers were enrolled. Patients who returned self-report questionnaires were included (*N* = 2246). Patients were between 18 and 85 years old. Mean age was 39.5 years (sd = 12.0).

### Assessments

#### Patient rated assessments

##### Quality of life

Patients completed the Manchester Short Assessment of Quality of Life (MANSA) [18]. The MANSA is designed to assess the quality of life of patients with mental disorders and has satisfactory reliability and validity for this patient group [18,19]. The MANSA comprises four objective and 12 subjective items and the subjective items were used in this study. The items are designed to assess the satisfaction the patients derive from the following domains: life as a whole, job, financial situation, number and quality of friendships, leisure activities, accommodation, personal safety, people the patient lives with (or living alone), sex life, relationship with family, physical health, and mental health. The MANSA is rated on a 7-point Likert scale (1 = couldn’t be worse, 7 = couldn’t be better). The score used in this study is the mean of the 12 items. 344 patients did not complete the MANSA. Cronbach’s alpha was 0.87 for the included sample.

##### Symptom severity

Patients completed the Symptom Checklist – 25 (SCL-25) in 2002, and the Symptom Checklist – 10 (SCL-10) in 2005. Both are short versions of the SCL-90-R, which has been extensively used in research and clinical settings, and the Norwegian translations have satisfactory reliability and validity [20]. The SCL-25 consists of 25 items, and the SCL-10 consists of 10 items, describing severity of psychiatric symptoms rated on a 4-point scale (1 = not at all, 2 = a little severe, 3 = quite severe, 4 = very severe). All items on the SCL-10 are present in the SCL-25 and only the SCL-10 items were used in the current study. 101 patients did not complete the SCL-10 and the Cronbach’s alpha was 0.89 for the included sample. A mean score of 1.85 or higher indicates mental disturbance on the SCL-10 [20].

##### Benefit from treatment

Patients evaluated the degree of benefit they had received from treatment at the time of the study. The patients rated four domains using a 5-point Likert scale (1 = very little benefit, 2 = rather little, 3 = neither little nor much, 4 = quite much, 5 = very much benefit). The four domains were: symptom reduction, symptom management skills, practical day-to-day functioning, and ability to work. 154 patients did not complete the treatment evaluation and Cronbach’s alpha for the four domains was 0.79 for the included sample.

##### Sleep disturbance

Three items measure sleep disturbance on the SCL-90R. On the SCL-25 and SCL-10, these three items have been reduced to one item measuring severity of sleep disturbance the past 14 days on a 1 to 4 likert scale (How much have sleep problems disturbed you the past 14 days: 1 = not at all, 2 = a little severe, 3 = quite severe, 4 = very severe disturbance). This item was used to assess patient rated level of sleep disturbance. The use of such a simple dimensional measure of sleep quality is in accordance with the proposals for field trials made by the DSM-5 Sleep-Wake Disorders Workgroup and Advisors [21].

#### Clinician rated assessments

##### Disturbance severity

The clinicians used the Health of Nations Outcome Scales (HoNOS) to evaluate the patients’ level of disturbance severity. The HoNOS is a 12-item clinician rated scale designed to measure the health and social functioning of patients with mental disorders. It has been widely used for patients in mental health care settings, and a review of the studies on the psychometric properties concluded that the HoNOS has adequate reliability and validity [22] The HoNOS has four subscales, behavior problems, cognitive impairment, symptoms and social functioning, and all items are rated on a 0 – 4 scale (0 = no problem, 1 = minor problem requiring no action, 2 = mild problem but definitely present, 3 = moderately severe problem, 4 = severe to very severe problem) [23]. The sum score of the HoNOS was used to assess the level of disturbance severity. Because the instructions on how to code item 8 (symptoms) where somewhat different in the two data-sets, this item was omitted from the sum score in the main analyses. The sum score of the HoNOS was 10.2 (SD = 4.9) for all 12 items on the HoNOS. Please see Table 1 for the sum score the HoNOS with item 8 omitted. There were missing items on 442 patients on the HoNOS.

**Table 1:** Mean scores on the independent variables for the patients from eight public mental health care centres who were included in the study

	**Mean**	**(SD)**	***n***	***Response***^***+***^***(%)***
*Patient rated variables*				
Manchester Short Assessment of Quality of Life	4.2	(0.9)	1902	84.7
Symptom Checklist – 10	2.4	(0.7)	2124	94.6
Benefit from Treatment	2.9	(1.1)	1657	91.5
*Clinician rated variables*				
Health of Nations Outcome Scales sum^++^	7.8	(4.5)	1804	80.3
Global Assessment of Functioning - Function	55.0	(10.6)	1945	86.6
Global Assessment of Functioning - Symptoms	54.6	(12.3)	1945	86.6
Benefit from Treatment	4.1	(1.0)	1721	95.0

*Notes*.

^+^ Of included patients.

^++^ Sum score omitting item 8.

##### Global assessment of functioning

The clinicians used a split version of the Global Assessment of Functioning Scale. The Global Assessment of Functioning Scale is described in Axis V of the Diagnostic and Statistical Manual of Mental Disorders – IV (DSM-IV) [4]. The split version is divided into one function score (GAF-F) and one symptom score (GAF-S). This has been done because of skepticism concerning the use of a single scale to measure both level of social and occupational function and severity of psychiatric symptoms [24]. The split version is reliable and consistent across raters [25].

##### Functioning

The GAF-F is a clinician rated 0–100 scale measuring the social, occupational, and psychological functioning of adults, e.g., how well or adaptively one is meeting various problems in living. There were missing data for 301 patients on the GAF-F.

##### Symptom severity

The GAF-S was used to assess the patients’ overall level of symptom severity. The GAF-S is a clinician rated 0–100 scale measuring the level of symptomatic distress [25]. There were missing data for 301 patients on the GAF-S.

##### Improvement from treatment

Clinicians rated the level of improvement from the beginning of treatment to the point of data collection on three domains using a 7-point Likert scale (1 = much worse, 2 = a little worse, 3 = no change, 4 = a little better, 5 = a bit better, 6 = much better, 7 = very much better). The three domains were: psychiatric symptoms, practical day-to-day functioning, and ability to work. Treatment improvement was not evaluated for 188 patients and Cronbach’s alpha for the three domains was 0.81 for the included sample.

#### Primary diagnoses

Patients were assigned one primary and up to two additional ICD-10 [5] diagnoses according to ordinary clinical practice. We used the primary diagnosis in this study. Diagnoses were first grouped into the ten main diagnostic chapters of the ICD-10 chapter 5: F0 Organic, including symptomatic, mental disorders; F1 Mental and behavioral disorders due to psychoactive substance use; F2 Schizophrenia, schizotypal and delusional disorders; F3 Mood disorders; F4 Neurotic, stress-related and somatoform disorders; F5 Behavioral syndromes associated with physiological disturbances and physical factors; F6 Disorders of adult personality and behavior; F7 Mental retardation; F8 Disorders of psychological development; and F9 Behavioral and emotional disorders with onset usually occurring in childhood and adolescence. Because few patients had received any of the diagnosis of F0 Organic, F1 Substance related, F5 Behavioral Syndromes, F7 Retardation, F8 Developmental, and F9 Childhood disorders, these patients were grouped into one group entitled Other Disorders in the analyses (N = 175). 291 patients had not received an ICD-10 diagnosis of mental or behavioral disorder at the time of data collection. No patients had received a diagnosis of insomnia or any other sleep related diagnoses as a primary or comorbid diagnosis.

#### Treatment duration

The number of months the patients had been in treatment at the time of the study was 14.5 months (sd = 28.7), whereas the median duration of treatment was 7 months.

#### Types of care

Patients were either receiving treatment as in-patients or out patients.

### Statistical analyses

The dependent variables in the statistical analyses were quality of life, patient rated symptom severity, patient rated benefit from treatment, disorder severity, level of functioning, clinician rated level of symptom severity and clinician rated benefit from treatment.

To examine differences in level of sleep disturbance in the different primary diagnostic groups we performed a one-way analysis of variance (ANOVA). To test if there were differences in levels of sleep disturbance between type of care, and men and women we performed two independent t-tests.

Six of the seven dependent variables were normally distributed and used in six hierarchical multiple regression analyses to test if sleep disturbance was related to the dependent variables independently of age and gender, time in treatment, type of care and primary diagnoses. We entered age and gender in step 1, time in treatment in step 2, type of care in step 3, primary diagnostic groups in step 4, sleep disturbance in step 5, and the interactions between sleep disturbance and the primary diagnostic groups in step 6. Because the variable “Clinician rated improvement from treatment” was not normally distributed, and could not be normalized, we dichotomized this variable and performed logistic regression analysis to test if sleep disturbance was related to good or poor clinician rated improvement from treatment. Patients with worsening or no improvement from treatment were classified as having “poor outcome” and patients with various degrees of improvement were classified as having “good outcome”. The logistic regression analysis was performed with the same hierarchical structure as the linear regression analyses.

Because of the number of statistical analyses, we Bonferroni corrected the level of significance to *p* < 0.007. Missing data was handled using listwise deletion. Mean score for the SCL was calculated omitting the sleep item. The statistical analyses were performed using PASW version 18 for Mac Os X.

### Ethics

The study was approved by the Regional Ethical Committee for Research in Health and by the Norwegian Data Inspectorate. The Directorate of Health and Social Affairs gave consent for the use of information from the health services.

## Results

### Descriptive and preliminary analyses

Diagnostic distribution, type of care and gender is reported in Table 2 along with the mean scores and standard deviations on sleep disturbance for each group.

**Table 2:** Descriptive and clinical data for patients from eight public mental health care centres included in the study

	**n**	**(%)**	**Mean Level of Sleep Disturbance**	**(sd)**
*Primary diagnoses*				
Schizophrenia	218	(9.7)	1.89	(0.98)
Mood disorders	799	(35.6)	2.39	(1.02)
Anxiety disorders	538	(24.0)	2.39	(1.02)
Personality disorders	225	(10.0)	2.39	(1.06)
Other disorders	175	(7.8)	2.39	(1.06)
No diagnosis	291	(13.0)	2.32	(1.06)
*Type of care*				
Out-patients	1754	(78.1)	2.34	(1.04)
In-patients	492	(21.9)	2.31	(1.01)
*Gender*				
Female	1127	(51.0)	2.36	(1.04)
Male	1081	(49.0)	2.29	(1.03)

Mean scores, standard deviations, and response rates on the dependent variables are shown in Table 1.

Patients with an ICD-10 diagnosis of Schizophrenia (chapter F20) reported significantly lower levels of sleep disturbance than other patients (F _(4, 1942)_ = 11.9, p < 0.0001). Levels of sleep disturbance were not different between patients in different types of care (t (2233) = 0.46, p = 0.64) or between men and women (t (2195) = 1.51, p = 0.13).

### Results from steps 1 – 4 of the regression analyses

The complete results from the seven regression analyses are shown in the Additional files. Please see Additional file 1: Table S1, Additional file 2: Table S2, Additional file 3: Table S3, Additional file 4: Table S4, Additional file 5: Table S5, Additional file 6: Table S6, Additional file 7: Table S7.Below is a summary of the significant results from steps 1 to 4 of the regression analyses.

Step 1. Older age was associated with higher quality of life (*β* = 0.10, *t* = 3.95, *p* < 0.0001).

Step 2. Longer duration of treatment was associated with higher degree of patient rated (*β* = 0.09, *t* = 3.60, *p* = 0.0003) and clinician rated (*B* = −0.02, *wald* = 36.9, *p* < 0.0001) improvement from treatment.

Step 3. Being an in-patient was associated with poorer quality of life (*β* = −0.10, *t* = 3.81, *p* = 0.0002), higher patient rated symptom severity (*β* = 0.09, *t* = 3.88, *p* = 0.0001), higher disorder severity (*β* = 0.17, *t* = 6.79, *p* < 0.0001), lower level of functioning (*β* = − 0.23, *t* = 9.97, *p* < 0.0001), higher clinician rated symptom severity (*β* = − 0.24, *t* = 10.3, *p* < 0.0001), and lower clinician rated benefit from treatment (*B* = −0.38, *wald* = 8.76, *p* = 0.003).

Step 4. Patients assigned a diagnosis of Schizophrenia had lower patient rated symptom severity (*β* = − 0.16, *t* = 2.93, *p* = 0.003), lower levels of functioning (*β* = − 0.24, *t* = 4.05, *p* = 0.0001), and higher levels of clinician rated symptom severity (*β* = −0.32, *t* = 5.66, *p* < 0.0001).

### Hypothesis testing

Level of sleep disturbance, entered in the fifth step of the regression analyses, was significantly and uniquely associated with all the seven dependent variables. See Table 3 for a summary of these results. The interactions between sleep disturbance and specific diagnoses, entered in step 6, were not significantly associated with any of the dependent variables.

**Table 3:** Summary of six linear hierarchical regression analyses and one logistic hierarchical regression analysis assessing the unique associations between sleep disturbance and the dependent variables adjusted for age, gender, time in treatment, type of care, and diagnoses for patients from eight public mental health care centres

**Dependent variables**	**Adj. R**^**2**^	**ΔR**^**2**^	**B**	**S.E. B**	**β**	**t**
Patient rated variables						
Quality of Life	0.12	0.08	−0.26	0.02	−0.29**	12.0
Symptom severity	0.20	0.17	0.29	0.02	0.42**	19.2
Benefit from treatment	0.02	0.01	−0.08	0.03	−0.08*	3.3
Clinician rated variables						
Disorder severity	0.11	0.05	0.97	0.10	0.22**	9.3
Level of functioning	0.13	0.02	−1.82	0.27	−0.15**	6.7
Symptom severity	0.17	0.02	−1.59	0.23	−0.16**	6.9
			B	S.E. B	Wald	OR
Benefit from treatment^+^			0.33	0.05	40.7**	1.39

*Notes.* * p = 0.001, ** p < 0.0001. ^+^Clinician rated benefit from treatment was tested using logistic regression analysis. Only sleep disturbance entered in the fifth step is shown in the table, except Adj. R^2^ which is the explained variance of all steps in the regression analyses. ΔR^2^ is the explained variance of sleep disturbance entered in the fifth step of the regression analyses.

Quality of Life = Mean score on the Manchester Assessment of Quality of Life. Patient rated Symptom Severity = The mean score of the Symptom Checklist (omitting the sleep item). Disorder Severity = The Sum Score of the Health of Nations Outcome Scales. Level of Functioning = The Global Assessment of Functioning Scale (split version) Function Subscale. Clinician rated Symptom Severity = The Global Assessment of Functioning Scale (split version) Symptom Subscale.

## Discussion

### Main findings

We found that higher levels of sleep disturbance were associated with significantly lower quality of life, higher distress and functional impairment, and less benefit from treatment for patients in mental health care, independently of their primary diagnosis. To our knowledge, it is the first time this has been shown in a large sample of patients representative of public mental health care clinics. These results might encourage clinicians to assess and provide specific treatment for sleep disturbance to patients with mental disorders as this might improve treatment results.

The DSM definition of a mental disorder is “a behavior or psychological syndrome that is associated with present distress or disability” [4]. Our results are in line with this definition, and with the recommendation from the NIH [16], and the suggested change in the DSM-5 [17], that sleep disturbance in patients with mental disorders may be regarded as a standalone therapeutic entity comorbid to the primary mental disorder.

### Interpretation in relation to previous research

The present study demonstrates associations between sleep disturbance and patient rated quality of life and clinician rated disorder severity and level of functioning. Quality of life has been defined as “a concept encompassing a broad range of physical and psychological characteristics and limitations which describe an individual’s ability to function and to derive satisfaction from doing so” [26]. The MANSA measures the patients’ satisfaction with social, professional, physical and emotional factors. The HoNOS measures mental and social functioning, whereas the GAF measures the general level of functioning. As such, our combined results, encompasses these broad features of quality of life laid out in the above definition. In 2005 the NIH recommended that future studies should focus on the association between quality of life and sleep disturbance [16], and emerging reports from the last years describe individuals with sleep disturbance to have poor quality of life [26,27] independently of somatic complaints [27,28] and also when the sleep disturbance is comorbid to depression [29]. Thus our study extends the current body of research by showing that sleep disturbance is associated with poor subjective quality of life and functioning in all patient groups in mental health care.

An important finding from the current study was that higher levels of sleep disturbance were strongly associated with higher levels of psychiatric symptom severity as measured by both clinicians and patients. This is similar to previous findings where patients with primary insomnia report high levels of emotional distress [30] and more negative affect [31]. Depressed patients with insomnia also report higher levels of symptom severity than depressed patients without insomnia [29,32], and the current study extends these findings to patients with other mental disorders. In sum, it is becoming more likely that sleep disturbance has a unique contribution to patients’ level of psychiatric symptom severity, their daily functioning and their quality of life that cannot be accounted for by the presence of other mental disorders.

The results from the present study also raise the question if sleep disturbance affects treatment outcome. We found that higher levels of sleep disturbance were associated with deriving less benefit from treatment as measured by both patients and clinicians. These results are in line with previous research on the impact of sleep disturbance on the treatment of depression. Untreated, sleep disturbance in depressed patients predicts poorer response to psychotherapy [33] and is the most common residual symptom after successful treatment of depression [11,12]. On the other hand, providing specific treatment for insomnia for patients with major depression who are receiving anti-depressant medication can enhance the effect of the medication [14,34] and improve quality of life [35]. One study even found that only providing cognitive behavior therapy for insomnia (CBT-I) to patients with mild depression normalized depression scores in 87% of the patients [15]. Interestingly, a recent pilot trial of CBT-I in 15 patients with persistent persecutory delusions and comorbid insomnia gave very promising results. Treatment with CBT-I did not only lead to large improvements in the insomnia of these patients but were also associated with large improvements in persecutory delusions [36]. There is less knowledge about the clinical impact of sleep in the treatment of anxiety disorders. However, one study found that sleep does not improve after successful treatment of panic disorder [37]. Providing CBT-I for patients with Post Traumatic Stress Disorder (PTSD) can also improve both sleep quality and other PTSD symptoms including nightmare frequency [38]. Thus, from the body of research that has been conducted so far, it seems that treating sleep disturbance may enhance treatment outcome, whereas no treatment for sleep disturbance could result in poorer outcome. From the findings in the present study it would be interesting for future research to explore if providing specific treatment of sleep disturbance in any mental disorder can have similar positive results.

Indeed, an interesting finding from the current study was that sleep disturbance was equally common in all diagnostic groups with the exception of schizophrenia, where there was less. This is in line with a recent review highlighting sleep disturbance as a potential transdiagnostic mechanism across mental disorders [39]. From a neurobiological point of view, Harvey et al. proposes that there is a bidirectional relationship between sleep disturbance and emotion regulation that may account for how sleep disturbance and emotional distress are linked in mental disorders [39]. Our finding is also similar to the conclusions of a meta-analysis of polysomnographically measured sleep in psychiatric patients where no differences between diagnostic categories could be found [40]. The low frequency of self-reported sleep disturbance among patients suffering from schizophrenia both in the present study and an earlier study [41] might both be explained by selection of patients, problems with insight, and characteristics of the illness.

### Limitations

There are limitations to the study that should be noted. First, the treatment results were rated retrospectively using rating scales that had not previously been validated. Social desirability factors, expectancy effects, and the patients’ feedback might have biased the clinician rated improvement scores and it is worth noticing that, on average, the clinicians reported more favorable outcomes than the patients did. This might explain why the clinicians’ rated outcomes were not normally distributed. Second, there may be selection biases, as about two-thirds of the original sample of patients did not agree to have their self-report linked to the clinician- report. This could be an artifact of the procedures. The patients had to specifically indicate that they wanted to have their scores linked to their clinicians’ ratings, rather than having to indicate if they did not want to have their scores linked. This difference could have had a large impact on patient participation [42]. However, our sample of patients seems representative of patients in public mental health care settings compared to findings from similar samples in other countries using the same measures [18,20,23,43-45]. Third, in the current study, the limited explained variance on some measures may be due to the study design. The study was commissioned by the Norwegian department of health to evaluate the state of the National mental health care system and was not designed specifically to evaluate the effects of co-occurring sleep disturbance in patients with mental disorders. Fourth, the cross-sectional design of the study does not allow for conclusions about causality and we cannot elucidate the exact relationship between sleep disturbance and symptom severity. Moreover, the study is not able to identify the potential underlying mechanisms that might link sleep disturbance to various mental disorders [39]. Fifth, a single item measuring sleep disturbance was used. This means that we cannot discern the relative impact of sleep onset or sleep maintenance problems or if the patients experienced other kinds of sleep disturbance. Still, it is remarkable that a single item measuring sleep disturbance had a significant effect on all included dependent variables. Indeed, that a single item can be used to get significant results is in line with previous research and may be useful for clinicians [46]. The work-group for sleep disorders in the DSM-5 revision has recently called for data from clinical settings with simple dimensional measures of sleep quality [21]. To compensate for these limitations, future studies should have a prospective study design where measurements are done before, during, and after treatment using validated outcome measures and scales adjusting for social desirability.

## Conclusions

In mental health care settings, sleep disturbance has a unique association with quality of life, symptom severity, disorder severity, level of functioning, and benefit from treatment over and above the effects of age, gender, time in treatment, type of care, and primary diagnoses. Thus, sleep disturbance ought to be considered a stand-alone therapeutic entity rather than an epiphenomenon of existing diagnoses for patients receiving treatment in mental health care.

## Abbreviations

NIH: National institutes of health; DSM-5: Diagnostic and statistical manual of mental disorders, 5th edition; MANSA: The manchester short assessment of quality of life; SCL: The symptom checklist; HoNOS: The health of nations outcome scales; GAF – F: The global assessment of functioning scale – function subscale; GAF – S: The global assessment of functioning scale – symptom subscale; DSM-IV: Diagnostic and statistical manual of mental disorders, 4th edition; ICD-10: The international classification of disorders, 10th edition; ANOVA: Analysis of variance; CBT-I: Cognitive behavioral therapy for insomnia; PTSD: Post traumatic stress disorder.

## Competing interests

All authors declare that we do not have any conflicts of interest.

## Authors’ contributions

HK conceptualized the report, did the data analyses, data interpretation, and wrote the report. BH, KL, GM, and TCS conceptualized the report, did data interpretation, and critical appraisals of the report. RG and TR made the study designs, conducted the data collections, and made critical appraisals of the report. All authors read and approved the final manuscript.

## Pre-publication history

The pre-publication history for this paper can be accessed here:

http://www.biomedcentral.com/1471-244X/12/179/prepub

## Supplementary Material

Additional file 1 **Table S1.** The hierarchical regression analysis of predictors of quality of life^a^ for patients in eight mental healthcare centers in Norway. Click here for file

Additional file 2 **Table S2.** The hierarchical regression analysis of predictors of patient rated symptom severity^a^ for patients in eight mental healthcare centers in Norway. Click here for file

Additional file 3 **Table S3.** The hierarchical regression analysis of predictors of patient rated benefit from treatment for patients in eight mental healthcare centers in Norway. Click here for file

Additional file 4 **Table S4.** The hierarchical regression analysis of predictors of disorder severity^a^ for patients in eight mental healthcare centers in Norway. Click here for file

Additional file 5 **Table S5.** The hierarchical regression analysis of predictors of level of functioning^a^ for patients in eight mental healthcare centers in Norway. Click here for file

Additional file 6 **Table S6.** The hierarchical regression analysis of predictors of clinician rated level of symptom severity^a^ for patients in eight mental healthcare centers in Norway. Click here for file

Additional file 7 **Table S7.** The hierarchical regression analysis of predictors of clinician rated level of improvement for patients in eight mental healthcare centers in Norway. Click here for file
