# Supplementary material for: Impact of sleep disturbance on patients in treatment for mental disorders
Source: BMC Psychiatry. 2012 Oct 29;12:179. doi: 10.1186/1471-244X-12-179 (PMC3505143; doi:10.1186/1471-244X-12-179)
Supplement: Additional file 1 — Table S1. The hierarchical regression analysis of predictors of quality of lifea for patients in eight mental healthcare centers in Norway. [file 1471-244X-12-179-S1.doc]

**Supplement table 1. The hierarchical regression analysis of predictors of quality of lif­e^a^ for patients in eight mental healthcare centers in Norway.**

| Step | | Independent variables | B | S.E. B | β | *t* | *p* |
| --- | --- | --- | --- | --- | --- | --- | --- |
| 1 |  | | | | | | |
|  | Age | | 0.01 | 0.00 | 0.10 | 3.95 | 10^-4^ |
|  | Gender | | 0.01 | 0.02 | 0.01 | 0.45 | 0.65 |
| 2 |  | | | | | | |
|  | Time in Treatment | | 0.00 | 0.00 | 0.01 | 0.22 | 0.83 |
| 3 |  | | | | | | |
|  | Type of Care | | -0.11 | 0.03 | -0.10 | 3.81 | 0.0002 |
| 4 |  | | | | | | |
|  | Schizophrenia | | 0.15 | 0.09 | 0.10 | 1.63 | 0.10 |
|  | Affective Disorders | | 0.06 | 0.09 | 0.06 | 0.66 | 0.51 |
|  | Anxiety Disorders | | 0.09 | 0.09 | 0.09 | 1.00 | 0.32 |
|  | Personality Disorders | | -0.09 | 0.09 | -0.06 | 0.94 | 0.35 |
|  | Other Diagnoses | | 0.10 | 0.09 | 0.06 | 1.07 | 0.28 |
| 5 |  | | | | | | |
|  | Sleep disturbance | | -0.26 | 0.02 | -0.29 | 11.98 | 10^-31^ |
| 6 |  | | | | | | |
|  | Sleep Disturbance X Schizophrenia | | 0.05 | 0.09 | 0.09 | 0.57 | 0.57 |
|  | Sleep Disturbance X Affective Disorders | | -0.03 | 0.08 | -0.09 | 0.42 | 0.67 |
|  | Sleep Disturbance X Anxiety Disorders | | -0.03 | 0.08 | -0.07 | 0.35 | 0.73 |
|  | Sleep Disturbance X Personality Disorders | | 0.01 | 0.09 | 0.02 | 0.14 | 0.89 |
|  | Sleep Disturbance X Other Disorders | | -0.06 | 0.09 | -0.11 | 0.71 | 0.48 |
| a. Dependent Variable: The Manchester Short Assessment of Quality of Life (MANSA) | | | | | | | |
