# Supplementary material for: Impact of sleep disturbance on patients in treatment for mental disorders
Source: BMC Psychiatry. 2012 Oct 29;12:179. doi: 10.1186/1471-244X-12-179 (PMC3505143; doi:10.1186/1471-244X-12-179)
Supplement: Additional file 2 — Table S2. The hierarchical regression analysis of predictors of patient rated symptom severitya for patients in eight mental healthcare centers in Norway. [file 1471-244X-12-179-S2.doc]

**Supplement table 2. Hierarchical regression analysis of predictors of patient rated symptom severity^a^** for patients in eight mental healthcare centers in Norway.

|  | | | | | | | |
| --- | --- | --- | --- | --- | --- | --- | --- |
| Step | | Independent variables | B | S.E. B | β | *t* | *p* |
| 1 |  | | | | | | |
|  | Age | | 0.00 | 0.00 | 0.00 | 0.16 | 0.87 |
|  | Gender | | -0.03 | 0.02 | -0.05 | 1.87 | 0.06 |
| 2 |  | | | | | | |
|  | Time in Treatment | | 0.00 | 0.00 | -0.05 | 1.87 | 0.06 |
| 3 |  | | | | | | |
|  | Type of Care | | 0.08 | 0.02 | 0.09 | 3.88 | 0.0001 |
| 4 |  | | | | | | |
|  | Schizophrenia | | -0.18 | 0.06 | -0.16 | 2.93 | 0.003 |
|  | Affective Disorders | | -0.04 | 0.06 | -0.05 | 0.66 | 0.51 |
|  | Anxiety Disorders | | -0.02 | 0.06 | -0.02 | 0.28 | 0.78 |
|  | Personality Disorders | | 0.03 | 0.06 | 0.02 | 0.44 | 0.66 |
|  | Other Diagnoses | | -0.05 | 0.06 | -0.04 | 0.75 | 0.46 |
| 5 |  | | | | | | |
|  | Sleep disturbance | | 0.29 | 0.02 | 0.42 | 19.15 | 10^-71^ |
| 6 |  | | | | | | |
|  | Sleep Disturbance X Schizophrenia | | -0.02 | 0.06 | -0.05 | 0.36 | 0.72 |
|  | Sleep Disturbance X Affective Disorders | | -0.02 | 0.05 | -0.06 | 0.30 | 0.77 |
|  | Sleep Disturbance X Anxiety Disorders | | -0.03 | 0.05 | -0.10 | 0.55 | 0.58 |
|  | Sleep Disturbance X Personality Disorders | | -0.02 | 0.06 | -0.06 | 0.39 | 0.70 |
|  | Sleep Disturbance X Other Disorders | | 0.00 | 0.06 | 0.01 | 0.05 | 0.96 |
| a. Dependent Variable: The Symprom Checklist (SCL) | | | | | | | |
