# Supplementary material for: Impact of sleep disturbance on patients in treatment for mental disorders
Source: BMC Psychiatry. 2012 Oct 29;12:179. doi: 10.1186/1471-244X-12-179 (PMC3505143; doi:10.1186/1471-244X-12-179)
Supplement: Additional file 3 — Table S3. The hierarchical regression analysis of predictors of patient rated benefit from treatment for patients in eight mental healthcare centers in Norway. [file 1471-244X-12-179-S3.doc]

**Supplement table 3. Hierarchical regression analysis of predictors of patient rated benefit from treatment for patients in eight mental healthcare centers in Norway.**

| Step | | Independent variables | B | S.E. B | Β | *t* | *p* |
| --- | --- | --- | --- | --- | --- | --- | --- |
| 1 |  | | | | | | |
|  | Age | | 0.00 | 0.00 | 0.05 | 2.20 | 0.03 |
|  | Gender | | 0.06 | 0.03 | 0.06 | 2.31 | 0.02 |
| 2 |  | | | | | | |
|  | Time in Treatment | | 0.00 | 0.00 | 0.09 | 3.60 | 0.0003 |
| 3 |  | | | | | | |
|  | Type of Care | | 0.00 | 0.03 | 0.00 | 0.03 | 0.97 |
| 4 |  | | | | | | |
|  | Schizophrenia | | 0.16 | 0.10 | 0.10 | 1.61 | 0.11 |
|  | Affective Disorders | | 0.06 | 0.09 | 0.06 | 0.68 | 0.50 |
|  | Anxiety Disorders | | 0.10 | 0.09 | 0.08 | 1.05 | 0.29 |
|  | Personality Disorders | | -0.02 | 0.10 | -0.01 | 0.18 | 0.85 |
|  | Other Diagnoses | | 0.09 | 0.10 | 0.04 | 0.83 | 0.40 |
| 5 |  | | | | | | |
|  | Sleep disturbance | | -0.08 | 0.03 | -0.08 | 3.27 | 0.001 |
| 6 |  | | | | | | |
|  | Sleep Disturbance X Schizophrenia | | -0.12 | 0.10 | -0.19 | 1.24 | 0.22 |
|  | Sleep Disturbance X Affective Disorders | | -0.15 | 0.09 | -0.35 | 1.61 | 0.11 |
|  | Sleep Disturbance X Anxiety Disorders | | -0.13 | 0.09 | -0.28 | 1.39 | 0.17 |
|  | Sleep Disturbance X Personality Disorders | | -0.09 | 0.10 | -0.15 | 0.88 | 0.38 |
|  | Sleep Disturbance X Other Disorders | | -0.03 | 0.10 | -0.05 | 0.33 | 0.74 |
