# Supplementary material for: Impact of sleep disturbance on patients in treatment for mental disorders
Source: BMC Psychiatry. 2012 Oct 29;12:179. doi: 10.1186/1471-244X-12-179 (PMC3505143; doi:10.1186/1471-244X-12-179)
Supplement: Additional file 4 — Table S4. The hierarchical regression analysis of predictors of disorder severitya for patients in eight mental healthcare centers in Norway. [file 1471-244X-12-179-S4.doc]

**Supplement table 4. Hierarchical regression analysis of predictors of disorder severity^a^ for patients in eight mental healthcare centers in Norway.**

| Step | | Independent variables | B | S.E. B | Β | *t* | *p* | |
| --- | --- | --- | --- | --- | --- | --- | --- | --- |
| 1 |  | | | | | | | |
|  | Age | | -0.02 | 0.01 | -0.06 | 2.22 | | 0.03 |
|  | Gender | | -0.07 | 0.11 | -0.01 | 0.60 | | 0.55 |
| 2 |  | | | | | | | |
|  | Time in Treatment | | 0.01 | 0.00 | 0.05 | 1.85 | | 0.06 |
| 3 |  | | | | | | | |
|  | Type of Care | | 0.91 | 0.13 | 0.17 | 6.79 | | 10^-11^ |
| 4 |  | | | | | | | |
|  | Schizophrenia | | 1.15 | 0.43 | 0.16 | 2.67 | | 0.01 |
|  | Affective Disorders | | 0.41 | 0.41 | 0.09 | 1.01 | | 0.31 |
|  | Anxiety Disorders | | 0.09 | 0.41 | 0.02 | 0.23 | | 0.82 |
|  | Personality Disorders | | 0.91 | 0.43 | 0.13 | 2.14 | | 0.03 |
|  | Other Diagnoses | | 0.97 | 0.44 | 0.12 | 2.20 | | 0.03 |
| 5 |  | | | | | | | |
|  | Sleep disturbance | | 0.96 | 0.10 | 0.22 | 9.30 | | 10^-19^ |
| 6 |  | | | | | | | |
|  | Sleep Disturbance X Schizophrenia | | -0.08 | 0.42 | -0.03 | 0.20 | | 0.84 |
|  | Sleep Disturbance X Affective Disorders | | 0.32 | 0.40 | 0.18 | 0.81 | | 0.42 |
|  | Sleep Disturbance X Anxiety Disorders | | -0.01 | 0.40 | -0.01 | 0.03 | | 0.98 |
|  | Sleep Disturbance X Personality Disorders | | -0.13 | 0.42 | -0.05 | 0.31 | | 0.75 |
|  | Sleep Disturbance X Other Disorders | | 0.37 | 0.43 | 0.14 | 0.87 | | 0.39 |
| a. Dependent Variable: The Health of Nations Outcome Scales (HoNOS) | | | | | | | | |
