# Supplementary material for: Impact of sleep disturbance on patients in treatment for mental disorders
Source: BMC Psychiatry. 2012 Oct 29;12:179. doi: 10.1186/1471-244X-12-179 (PMC3505143; doi:10.1186/1471-244X-12-179)
Supplement: Additional file 5 — Table S5. The hierarchical regression analysis of predictors of level of functioninga for patients in eight mental healthcare centers in Norway. [file 1471-244X-12-179-S5.doc]

**Supplement table 5. Hierarchical regression analysis of predictors of level of functioning^a^ for patients in eight mental healthcare centers in Norway.**

| Step | | Independent variables | B | S.E. B | β | *t* | *p* |
| --- | --- | --- | --- | --- | --- | --- | --- |
| 1 |  | | | | | | |
|  | Age | | -0.03 | 0.02 | -0.03 | 1.34 | 0.18 |
|  | Gender | | -0.07 | 0.30 | -0.01 | 0.23 | 0.82 |
| 2 |  | | | | | | |
|  | Time in Treatment | | -0.01 | 0.01 | -0.01 | 0.59 | 0.56 |
| 3 |  | | | | | | |
|  | Type of Care | | -3.50 | 0.35 | -0.23 | 9.97 | 10^-23^ |
| 4 |  | | | | | | |
|  | Schizophrenia | | -4.62 | 1.14 | -0.24 | 4.05 | 0.0001 |
|  | Affective Disorders | | -0.37 | 1.08 | -0.03 | 0.34 | 0.73 |
|  | Anxiety Disorders | | 0.30 | 1.09 | 0.02 | 0.27 | 0.78 |
|  | Personality Disorders | | -2.14 | 1.14 | -0.11 | 1.88 | 0.06 |
|  | Other Diagnoses | | -1.36 | 1.17 | -0.06 | 1.16 | 0.24 |
| 5 |  | | | | | | |
|  | Sleep disturbance | | -1.82 | 0.27 | -0.15 | 6.73 | 10^-10^ |
| 6 |  | | | | | | |
|  | Sleep Disturbance X Schizophrenia | | 0.95 | 1.13 | 0.13 | 0.85 | 0.40 |
|  | Sleep Disturbance X Affective Disorders | | 0.34 | 1.06 | 0.07 | 0.32 | 0.75 |
|  | Sleep Disturbance X Anxiety Disorders | | 0.60 | 1.07 | 0.11 | 0.56 | 0.57 |
|  | Sleep Disturbance X Personality Disorders | | 1.24 | 1.11 | 0.18 | 1.11 | 0.27 |
|  | Sleep Disturbance X Other Disorders | | 0.53 | 1.14 | 0.07 | 0.47 | 0.64 |
| a. Dependent Variable: Global Assessment of Functioning – Function Subscale (GAF – F) | | | | | | | |
