# Supplementary material for: Impact of sleep disturbance on patients in treatment for mental disorders
Source: BMC Psychiatry. 2012 Oct 29;12:179. doi: 10.1186/1471-244X-12-179 (PMC3505143; doi:10.1186/1471-244X-12-179)
Supplement: Additional file 6 — Table S6. The hierarchical regression analysis of predictors of clinician rated level of symptom severitya for patients in eight mental healthcare centers in Norway. [file 1471-244X-12-179-S6.doc]

**Supplement table 6. Hierarchical regression analysis of predictors of clinician rated level of symptom severity^a^ for patients in eight mental healthcare centers in Norway.**

| Step | | Independent variables | B | S.E. B | β | *t* | *p* |
| --- | --- | --- | --- | --- | --- | --- | --- |
| 1 |  | | | | | | |
|  | Age | | 0.00 | 0.02 | 0.00 | 0.18 | 0.86 |
|  | Gender | | -0.11 | 0.26 | -0.01 | 0.43 | 0.67 |
| 2 |  | | | | | | |
|  | Time in Treatment | | -0.01 | 0.01 | -0.02 | 0.95 | 0.34 |
| 3 |  | | | | | | |
|  | Type of Care | | -3.13 | 0.30 | -0.24 | 10.30 | 10^-23^ |
| 4 |  | | | | | | |
|  | Schizophrenia | | -5.50 | 0.97 | -0.32 | 5.66 | 10^-7^ |
|  | Affective Disorders | | -0.59 | 0.92 | -0.05 | 0.64 | 0.52 |
|  | Anxiety Disorders | | -0.38 | 0.93 | -0.03 | 0.40 | 0.69 |
|  | Personality Disorders | | -2.34 | 0.97 | -0.14 | 2.42 | 0.02 |
|  | Other Diagnoses | | -0.96 | 1.00 | -0.05 | 0.97 | 0.33 |
| 5 |  | | | | | | |
|  | Sleep disturbance | | -1.59 | 0.23 | -0.15 | 6.95 | 10^-11^ |
| 6 |  | | | | | | |
|  | Sleep Disturbance X Schizophrenia | | 2.10 | 0.96 | 0.33 | 2.20 | 0.03 |
|  | Sleep Disturbance X Affective Disorders | | 1.33 | 0.90 | 0.31 | 1.47 | 0.14 |
|  | Sleep Disturbance X Anxiety Disorders | | 1.67 | 0.91 | 0.37 | 1.83 | 0.07 |
|  | Sleep Disturbance X Personality Disorders | | 1.70 | 0.94 | 0.29 | 1.80 | 0.07 |
|  | Sleep Disturbance X Other Disorders | | 1.17 | 0.97 | 0.18 | 1.21 | 0.23 |
| a. Dependent Variable: Global Assessment of Functioning – Symptom Subscale (GAF – S) | | | | | | | |
