# Supplementary material for: Impact of sleep disturbance on patients in treatment for mental disorders
Source: BMC Psychiatry. 2012 Oct 29;12:179. doi: 10.1186/1471-244X-12-179 (PMC3505143; doi:10.1186/1471-244X-12-179)
Supplement: Additional file 7 — Table S7. The hierarchical regression analysis of predictors of clinician rated level of improvement for patients in eight mental healthcare centers in Norway. [file 1471-244X-12-179-S7.doc]

**Supplement table 7. Hierarchical logistic regression analysis of predictors of clinician rated level of improvement for patients in eight mental healthcare centers in Norway.**

| Step | | Independent variables | B | S.E. B | Wald | *OR* | *p* |
| --- | --- | --- | --- | --- | --- | --- | --- |
| 1 |  | | | | | | |
|  | Age | | 0.00 | 0.00 | 0.23 | 1.00 | 0.63 |
|  | Gender | | -0.02 | 0.10 | 0.03 | 0.98 | 0.86 |
| 2 |  | | | | | | |
|  | Time in Treatment | | -0.02 | 0.00 | 36.9 | 0.98 | 10^-9^ |
| 3 |  | | | | | | |
|  | Type of Care | | -0.38 | 0.13 | 8.76 | 0.69 | 0.003 |
| 4 |  | | | | | | |
|  | Schizophrenia | | -0.09 | 0.41 | 0.05 | 0.92 | 0.83 |
|  | Affective Disorders | | 0.21 | 0.38 | 0.32 | 1.24 | 0.58 |
|  | Anxiety Disorders | | 0.31 | 0.38 | 0.65 | 1.36 | 0.42 |
|  | Personality Disorders | | 0.20 | 0.40 | 0.26 | 1.22 | 0.61 |
|  | Other Diagnoses | | 0.04 | 1.53 | 0.13 | 1.72 | 0.72 |
| 5 |  | | | | | | |
|  | Sleep disturbance | | -0.33 | 0.05 | 40.8 | 1.39 | 10^-9^ |
| 6 |  | | | | | | |
|  | Sleep Disturbance X Schizophrenia | | -0.02 | 0.21 | 0.01 | 0.98 | 0.92 |
|  | Sleep Disturbance X Affective Disorders | | 0.05 | 0.19 | 0.07 | 1.05 | 0.80 |
|  | Sleep Disturbance X Anxiety Disorders | | 0.06 | 0.19 | 0.08 | 1.06 | 0.78 |
|  | Sleep Disturbance X Personality Disorders | | 0.16 | 0.20 | 0.62 | 1.17 | 0.71 |
|  | Sleep Disturbance X Other Disorders | | -0.08 | 0.21 | 0.14 | 0.93 | 0.71 |
